# *FGFR3*, *HRAS*, *KRAS*, *NRAS* and *PIK3CA* Mutations in Bladder Cancer and Their Potential as Biomarkers for Surveillance and Therapy

**DOI:** 10.1371/journal.pone.0013821

**Published:** 2010-11-03

**Authors:** Lucie C. Kompier, Irene Lurkin, Madelon N. M. van der Aa, Bas W. G. van Rhijn, Theo H. van der Kwast, Ellen C. Zwarthoff

**Affiliations:** 1 Department of Pathology, Erasmus MC, Rotterdam, The Netherlands; 2 Department of Urology, University Health Network, Toronto General Hospital, Toronto, Canada; 3 Department of Laboratory Medicine and Pathobiology, University Health Network, Toronto General Hospital, Toronto, Canada; Cleveland Clinic, United States of America

## Abstract

**Background:**

Fifty percent of patients with muscle–invasive bladder cancer (MI-BC) die from their disease and current chemotherapy treatment only marginally increases survival. Novel therapies targeting receptor tyrosine kinases or activated oncogenes may improve outcome. Hence, it is necessary to stratify patients based on mutations in relevant oncogenes. Patients with non-muscle-invasive bladder cancer (NMI-BC) have excellent survival, however two-thirds develop recurrences. Tumor specific mutations can be used to detect recurrences in urine assays, presenting a more patient-friendly diagnostic procedure than cystoscopy.

**Methodology/Principal Findings:**

To address these issues, we developed a mutation assay for the simultaneous detection of 19 possible mutations in the *HRAS*, *KRAS*, and *NRAS* genes. With this assay and mutation assays for the *FGFR3* and *PIK3CA* oncogenes, we screened primary bladder tumors of 257 patients and 184 recurrences from 54 patients. Additionally, in primary tumors p53 expression was obtained by immunohistochemistry. Of primary tumors 64% were mutant for *FGFR3*, 11% for *RAS*, 24% for *PIK3CA*, and 26% for p53. *FGFR3* mutations were mutually exclusive with *RAS* mutations (p = 0.001) and co-occurred with *PIK3CA* mutations (p* = *0.016). P53 overexpression was mutually exclusive with *PIK3CA* and *FGFR3* mutations (p≤0.029). Mutations in the *RAS* and *PIK3CA* genes were not predictors for recurrence-free, progression-free and disease-specific survival. In patients presenting with NMI-BC grade 3 and MI-BC, 33 and 36% of the primary tumors were mutant. In patients with low-grade NMI-BC, 88% of the primary tumors carried a mutation and 88% of the recurrences were mutant.

**Conclusions/Significance:**

The mutation assays present a companion diagnostic to define patients for targeted therapies. In addition, the assays are a potential biomarker to detect recurrences during surveillance. We showed that 88% of patients presenting with low-grade NMI-BC are eligible for such a follow-up. This may contribute to a reduction in the number of cystoscopical examinations.

## Introduction

Bladder cancer is the fifth most common cancer in the Western World [1]. Of the bladder tumors 15–20% presents as muscle-invasive disease (MI-BC), the remaining group as non-muscle-invasive tumors (NMI-BC). MI-BC is a devastating disease since over 50% of the patients will die from metastatic disease. Recently, new developments in targeted therapies using receptor tyrosine kinase inhibitors in other cancer types have inspired the possible treatment of patients with MI-BC with similar adjuvant agents [2]. For muscle-invasive bladder tumors FGFR3 targeted therapy is being considered [3], [4], [5], [6] and recently a Phase II study has started to investigate the efficacy of TKI258, an FGFR3 inhibitor, in patients with advanced urothelial cancer (www.ClinicalTrials.gov NCT00790426). Likewise, the epidermal growth factor receptor (EGFR) is frequently overexpressed in bladder cancer and might therefore be an important therapeutic target for MI-BC [7], [8], [9]. Currently, EGFR targeted treatment is being investigated for bladder cancer in several clinical trials (CALBG-90102, NCT00088946, NCT00380029). However, it has recently become clear that therapies targeting receptor tyrosine kinases might not be effective when tumors harbor mutations in the RAS-MAPK or PIK3CA-AKT pathways downstream of the receptors [10], [11], [12], [13], [14]. Nevertheless, agents inhibiting targets downstream in these pathways are in clinical trials. This suggests that screening bladder tumors for mutations in genes such as *FGFR3*, *RAS* and *PIK3CA* can be of importance for future therapy decisions. An easy test that can be implemented in the clinic will therefore be desirable.

For non-muscle-invasive bladder cancer (NMI-BC), the major problem is that after initial transurethral resection of the bladder (TURB), 50–70% of the patients develop multiple recurrences, with a chance of 10–20% that these will progress to MI-BC [15], [16], [17]. The recurrence risk and risk of progression necessitate a life-long follow-up by cystoscopy. The current standard is to perform a cystoscopy together with urine cytology every 3–4 months in the first 2 years and twice per year thereafter [18]. We have recently shown that in the Netherlands patients with NMI-BC undergo on average 20 cystoscopies during the first 9 years of follow-up [17], with a recurrence detected in only one of seven of these follow-up moments. For the USA and Europe with populations of 300 and 450 million, this would amount to 1 and 1.5 million cystoscopies yearly. Reduction of the number of cystoscopies by, for instance, a urine-based test is an important goal in order to improve quality-of-life [19], [20], [21]. In addition, it could lead to cost-reduction. Currently, bladder cancer is the most expensive cancer type for treatment per patient [22], [23]. However, cytology and many of the currently developed urine biomarkers have limited sensitivity for detection of low stage and grade tumors that form the main group that recur (reviewed in [24], [25], [26], [27], [28]).Therefore, there is a need for more sensitive urinary biomarkers that can be implemented into molecular diagnostic laboratories.

NMI-BC and MI-BC are genetically different [29], [30]. NMI-BC tumors are characterized by a high frequency of mutations in the *FGFR3* oncogene [31], [32] leading to constitutive activation of the RAS-MAPK pathway [33], [34], [35], [36], [37]. In MI-BC, mutations in the *TP53* gene prevail. Mutations in *FGFR3* and *TP53* are largely mutually exclusive suggesting that NMI-BC and MI-BC develop along different oncogenesis pathways [38], [39]. However, in stage pT1 tumors that invade the connective tissue layer underlying the urothelium, they often occur together [32], [38], [39]. Recently, somatic mutations in the *PIK3CA* oncogene, which encodes the catalytic subunit p110α of class-IA PI3-kinase, were described in 13–27% of bladder tumors [40], [41]. These mutations often coincided with *FGFR3* mutations. Mutations in the *RAS* oncogenes (*HRAS*, *KRAS*, *NRAS*) have also been found in 13% of bladder tumors and occurred in all stages and grades [41], [42]. They were mutually exclusive with *FGFR3* mutations. However, no data exist regarding the prognostic value, in terms of recurrence-free, progression-free and disease-specific survival, of *RAS* and *PIK3CA* mutations in bladder cancer either alone or in combination with other alterations. In some cancer types *PIK3CA* mutations have been associated with invasiveness and a worse prognosis [11], [43], [44], [45], [46]. On the other hand, there are examples of somatic mutations in benign skin lesions that do not progress [47], [48]. Regarding alterations in RAS and prognosis, in the past studies have been performed on the prognostic value of expression of RAS p21 protein, however the results were not concordant [49], [50], [51]. A recent study on the expression of *HRAS* in 48 pTa bladder tumors showed an inverse correlation of expression value with recurrence and progression [52]. However, there is no information on the prognostic value of mutations in the three *RAS* genes in bladder cancer.

We have recently shown that with *FGFR3* mutation analysis on urine samples from bladder cancer patients it was possible to detect recurrent tumors [53], [54]. The technical performance of the *FGFR3* mutation assay in these studies was excellent. Sixty-three percent of patients with NMI-BC are mutant for *FGFR3*. An additional goal of the present study was to investigate whether adding *RAS* and *PIK3CA* mutation analysis to the *FGFR3* mutation detection could potentially increase the percentage of patients that can be monitored using urine-based assays for these mutations. In addition, these assays could be of use in clinic to define patients who may benefit from targeted therapies. We have therefore developed a multiplex mutation assay for the detection of the most frequently occurring *HRAS*, *KRAS*, and *NRAS* mutations in bladder cancer. This assay is based on assays that we previously developed [53], [55], [56]. In our experience, these assays are sensitive, easy to perform and to interpret, and require only a few nanograms of DNA. The assays are also successful on DNA from formalin-fixed paraffin embedded (FFPE) tissue or urine [53], [54], [56].

We subsequently investigated the mutation spectrum of *FGFR3*, *HRAS*, *KRAS*, *NRAS* and *PIK3CA* in a large series of primary tumors of 257 patients with NMI-BC and MI-BC. Mutation status was also compared with p53 expression. The distribution of alterations in these six genes together has not been investigated in bladder tumors before. We further screened 184 recurrences of 54 patients to determine whether mutation status is consistent in recurrences with the purpose to examine if it is useful to start a longitudinal study on surveillance with these mutation assays for the detection of recurrent bladder cancer in voided urine specimens from patients. The frequency of mutations in *RAS* and *PIK3CA* in a longitudinal setting containing multiple recurrences of the same patient has also not yet been investigated before.

We conclude that the mutation assays present a companion diagnostic to stratify patients with MI-BC for targeted therapies. In addition, since 88% of the primary tumors of patients presenting with low-grade NMI-BC carried a mutation in the *FGFR3*, *RAS*, and/or *PIK3CA* genes and 88% of the recurrences were mutant, the assays are a potential tool to detect recurrences in DNA obtained from urine samples during surveillance, which may contribute to a reduction in the number of cystoscopical examinations and is worth to investigate.

## Methods

### Patient characteristics and ethics statement

Formalin-fixed paraffin embedded (FFPE) samples of primary tumors of an unselected group of 257 patients were obtained from Erasmus MC and St Franciscus Gasthuis, Rotterdam, the Netherlands. The tumor samples represent a subgroup of 286 samples that we previously described [32]. No tissue was available anymore for the 29 missing samples. The mean age of this group of patients at diagnosis was 65.7 years and male/female ratio was 3/1. Tumors were staged according to the Tumor Node Metastasis classification of 1997 [57] and grades were classified according to the World Health Organization criteria of 1973 [58]. Among the primary tumors, there were 166 pTa, 57 pT1, and 34 pT2-4 tumors. The grade distribution was 84 grade 1, 117 grade 2 and 56 grade 3 tumors. Of 54 patients that were treated at Erasmus MC and had developed one or more recurrences, formalin-fixed paraffin embedded tissue was collected from 184 consecutive recurrences. Clinical data of the tumors were obtained from patient's case history. Data were analyzed anonymously. FFPE samples were used according to the standards presented in “The Code for Proper Secondary Use of Human Tissues in the Netherlands” (http://www.federa.org/). Informed consent was therefore not needed to be obtained. This was approved by our Institutional Review Board. A recurrence was defined as a tumor removed at transurethral resection that subsequently was confirmed to be tumor tissue by a pathologist. Tumors removed within three months after transurethral resection were not considered a recurrence. Progression was defined as progression in stage and/or to grade 3. Disease-specific survival was defined as time from diagnosis to death of bladder cancer. Follow-up period was counted from the date of diagnosis. Censoring of patients occurred at their last clinical visit or when a patient died. Generally, the patients were followed and treated according to the guidelines of the European Association of Urology [18]. The medical-ethical committee of the Erasmus University and the University Hospital Rotterdam approved the study (METC 168.922/1998/55).

### DNA isolation and mutation analysis

Haematoxylin-eosin stained slides were used for histological diagnosis and served as templates for manual micro dissection from the respective tissue blocks. The dissected tumor samples contained a minimum of 70% tumor cells. Tumor samples were extracted from Formalin-fixed paraffin embedded tumor tissue by de-waxing with xylene and ethanol. DNA was isolated using DNeasy Tissue kit (Qiagen, Hilden, Germany), according to the protocol. P53, MIB-1 and p27^Kip1^ immunostaining was obtained from van Rhijn *et al.* [32].

**Figure 1:**
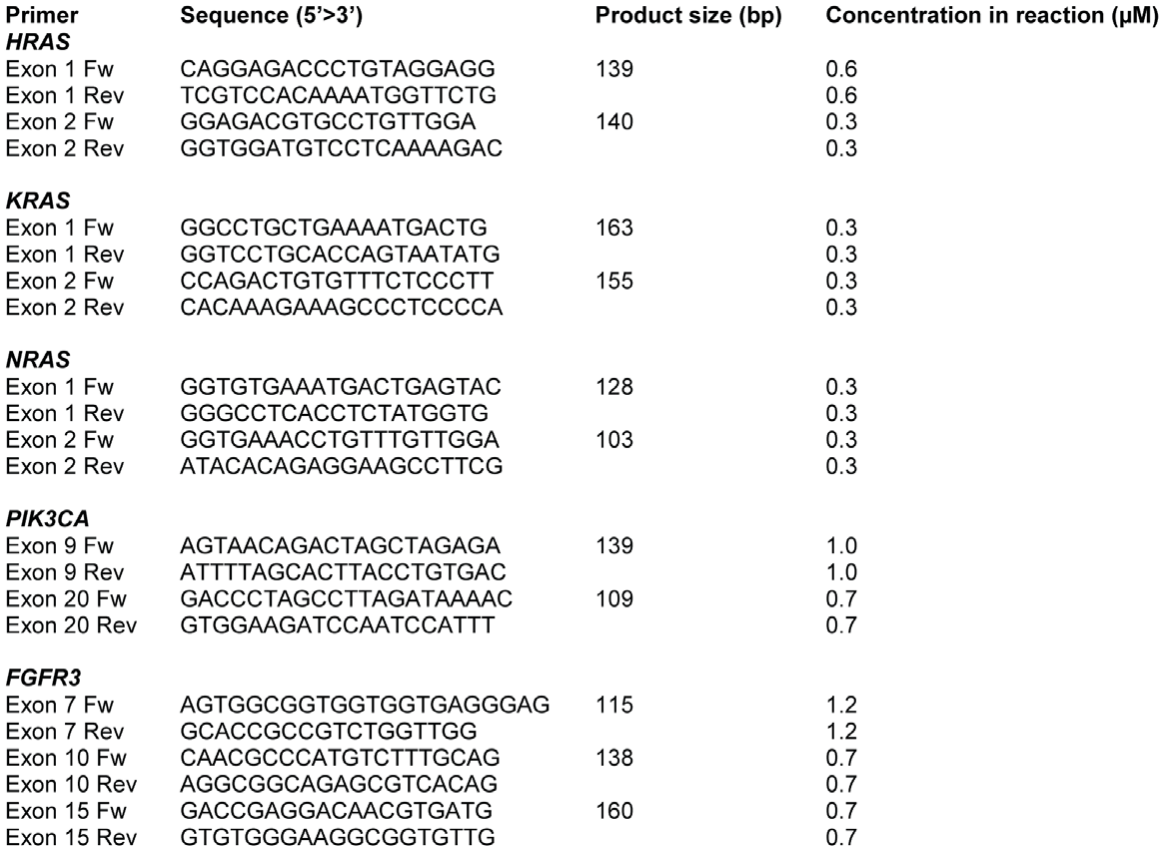
Primers used for multiplex amplification of *HRAS*, *KRAS*, *NRAS, PIK3CA*, and *FGFR3*.

**Figure 2:**
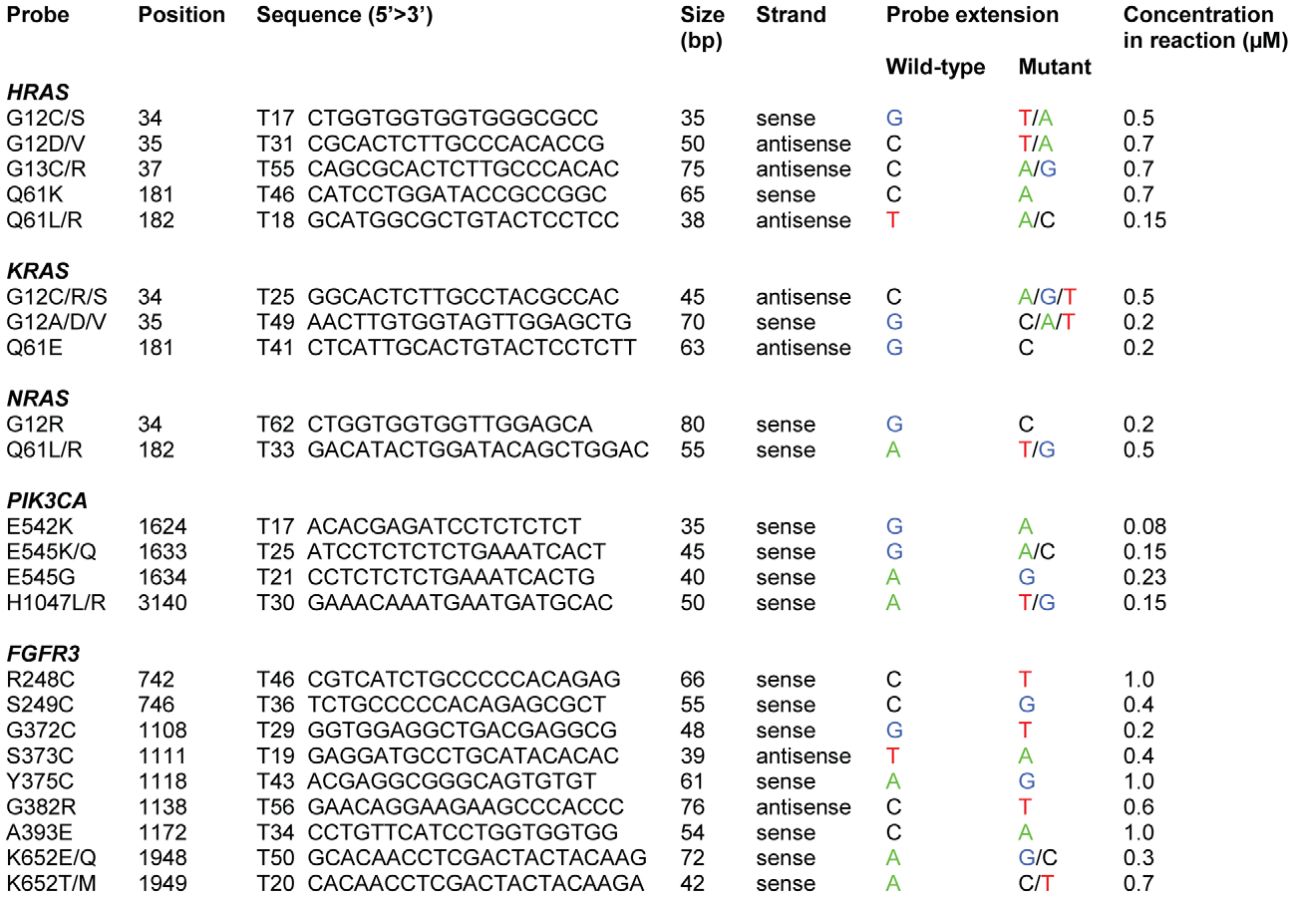
Probes for the detection of *HRAS*, *KRAS*, *NRAS*, *PIK3CA* and *FGFR3* mutations.

Primers for the multiplex RAS-BC assay were designed in such a way that the single strands of the PCR products contained as little potential secondary structure as possible in order to facilitate efficient annealing of the mutation detection probes. Primer design was further aimed at achieving identical annealing temperatures to allow simultaneous amplification of the relevant exons of the three *RAS* genes in one PCR reaction. Furthermore, the regions to be amplified were inspected for the presence of polymorphisms in the database of National Center for Biotechnology Information. No polymorphisms in these regions were observed. Mutation detection probes for multiplex detection of *HRAS*, *KRAS* and *NRAS* mutations were designed to anneal to either the forward or the reverse strand directly adjacent to the potential mutation site. With the assay, 19 possible mutations in 10 codons in the 3 *RAS* genes can be detected. Together they account for 96% of all somatic *HRAS*, *KRAS* and *NRAS* mutations found in urothelial cell carcinomas by the Sanger Institute (www.sanger.ac.uk/genetics/CGP/cosmic). To enable to distinguish the probes by size, poly(dT) tails of different lengths were added. All probes were designed to have similar annealing temperatures and were selected for the absence of secondary structures and base pairing with other probes. Primer and probe sequences and concentrations for the three multiplex mutation assays for *FGFR3, NRAS, HRAS, KRAS* and *PIK3CA* are depicted in Figure 1 and 2.

Each multiplex PCR reaction was performed in a total volume of 15 µl containing 0.17 mM dNTPs (Roche, Basel, Switzerland), 1.5 mM MgCl2, 5% glycerol (Fluka, Buchs SG, Switzerland), 0.3–1.2 µM of the appropriate primer combination (Invitrogen, Carlsbad, CA), 1× PCR buffer, and 0.5 units of Go Taq DNA polymerase (Promega, Madison, WI), using 5 ng genomic DNA as template. Thermal cycling consisted of initial denaturation at 95°C for 5 min, followed by 35 cycles of each 95°C for 45 sec, 55°C for 45 sec, and 72°C for 45 sec. The final elongation step was 72°C for 10 min. Unincorporated primers and deoxynucleotide triphosphates were removed from PCR products by addition of 2 units Exonuclease I (ExoI) and 3 units shrimp alkaline phosphatase (SAP, USB, Cleveland, Ohio USA).

PCR products were subsequently analyzed for mutations using probes for each of the possible mutation sites and the SNaPshot® Multiplex Kit (Applied Biosystems, Foster City, CA). The mutation detection reactions were performed in a total volume of 10 µl containing 2.5 µl of SNaPshot Multiplex Ready Reaction Mix, 2 µl BigDye sequencing buffer, 1 µl of probe mix and 1 µl of SAP/ExoI treated PCR product. Extension reactions consisting of 25 cycles of denaturation at 96°C for 10 sec and annealing/extension at 58.5°C for 40 sec, were performed in a thermal cycler. After extension, the excess of labeled dideoxynucleotide triphosphates was removed by treatment with 1 unit shrimp alkaline phosphatase at 37°C for 60 min and 72°C for 15 min. Extended primers were denatured at 95°C for 5 minutes and separated by capillary electrophoresis on an automatic sequencer (ABI PRISM 3130 XL Genetic Analyzer, Applied Biosystems, Foster City, CA), and the presence or absence of a mutation was indicated by the fluorescent label on the incorporated nucleotide. Details of colors of the mutant and wild-type peaks are given in Figure 2. Data were analyzed using GeneScan Analysis Software version 3.7 (Applied Biosystems) and GeneMarker Software version 1.7 (SoftGenetics LLC, State College, USA).

### Statistical analysis

Statistical analyses were performed using SPSS statistical package (version 15.0, SPSS, Inc., Chicago, IL, 2003). Differences were considered significant if p*<*0.05. The relationships between mutation status and pathological and clinical variables were analyzed by the Student's t-test, Chi-square test and two-sided Fisher exact tests. Recurrence-free, progression-free, and disease-specific survival by mutational status was analyzed using Kaplan-Meier curves. The two-sided log-rank test was performed to compare the curves.

## Results

### Bladder cancer specific RAS-BC mutation assay

**Figure 3:**
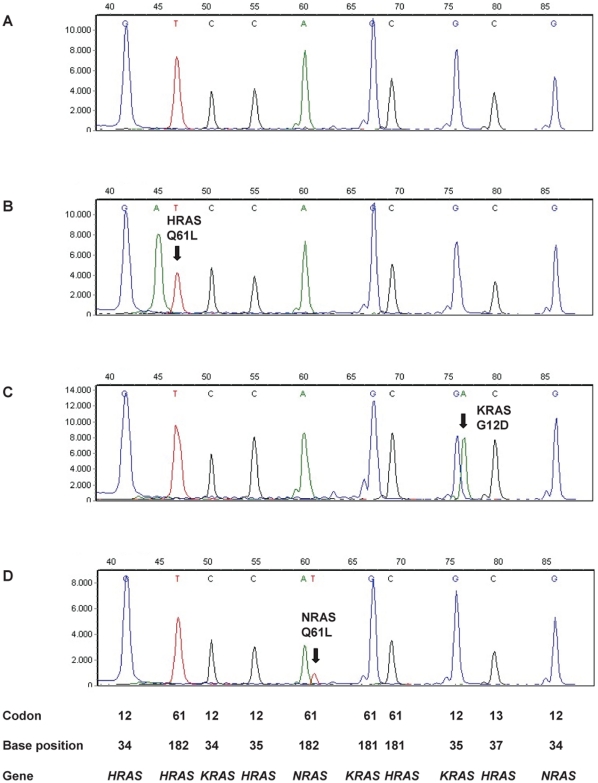
RAS-BC mutation assay. Panel A: wild-type sample, panels B–D: samples with mutations. Position of the interrogated codons, nucleotides and genes is depicted at the bottom.

Somatic mutations in the *HRAS*, *KRAS* and *NRAS* genes in bladder cancer affect codons 12, 13 and 61. In order to facilitate detection of *RAS* mutations we have developed a multiplex RAS-BC mutation assay that screens for 19 mutations simultaneously, representing 96% of all possible known mutations in the 3 *RAS* genes in bladder cancer (www.sanger.ac.uk/genetics/CGP/cosmic). The assay requires only a few nanograms of DNA and works well on DNA from formalin fixed tissue. Figure 3 shows examples of the RAS-BC assay with panel A representing the wild-type situation and with specific mutations depicted in panels B–D.

### Mutations in primary tumors

**Figure 4:**
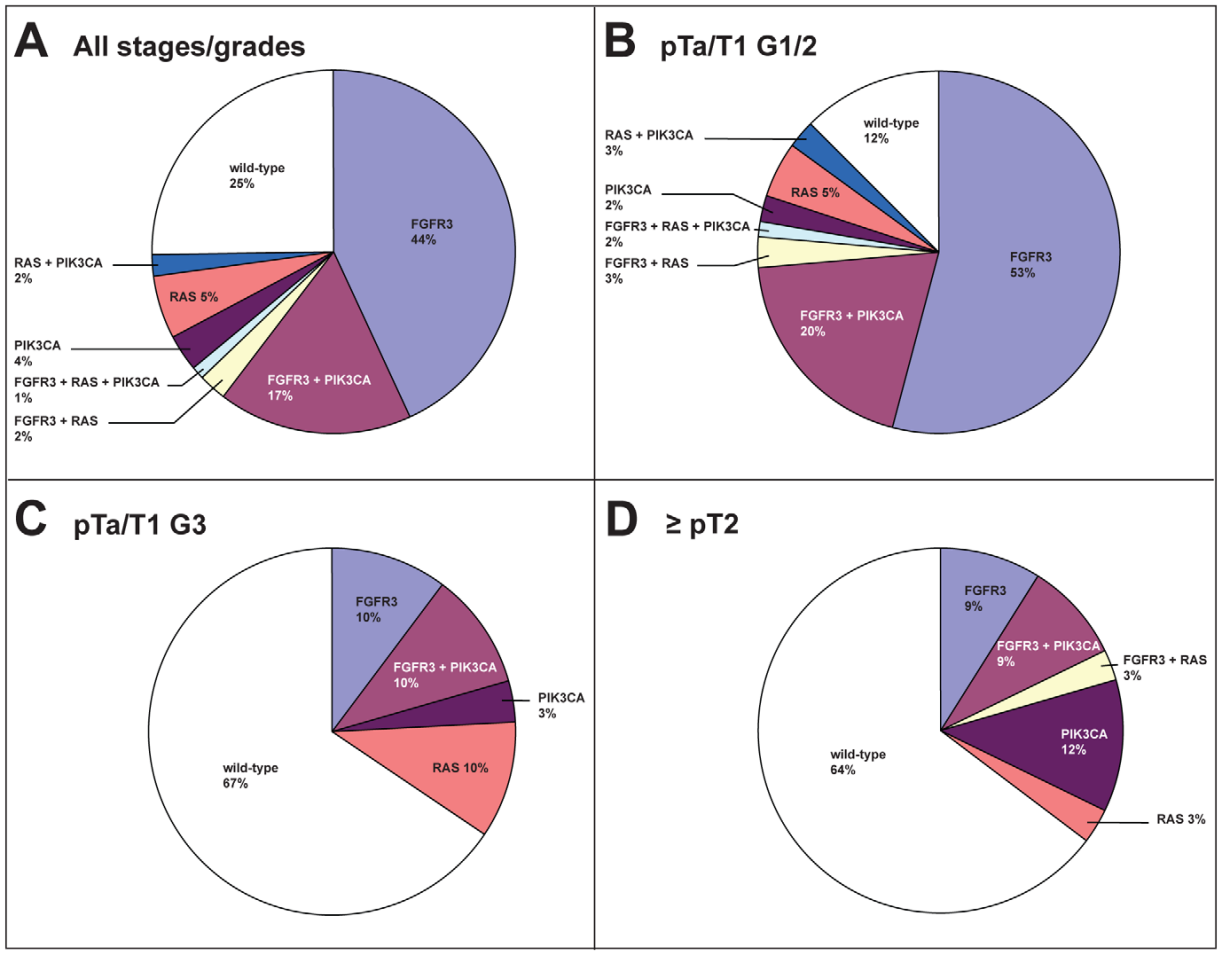
Frequencies of *FGFR3*, *PIK3CA* and *RAS* mutations in primary bladder tumors of 257 patients. Frequencies in all primary bladder tumors (A) (n = 257) and in specific tumor stages: pTa/T1-G1/G2 (B) (n = 194), pTa/T1G3 (C) (n = 29) and muscle-invasive tumors (D) (n = 34).

With the RAS-BC assay and mutation assays for *FGFR3* and *PIK3CA*, we screened primary bladder tumors of 257 patients for mutations (Figure 4A). Overall, 64% (164/257) of the tumors contained an *FGFR3* mutation, a total of 28 (11%) samples were mutant for one of the *RAS* genes and 61 (24%) harbored a *PIK3CA* mutation. Table 1 shows the type of the identified mutations. The most frequent *RAS* mutations were *KRAS* G12D and *HRAS* Q61R. *KRAS* and *HRAS* mutations occurred with equal frequency, whereas *NRAS* mutations were not frequent in bladder cancer. In the *PIK3CA* gene, the mutations occurred mostly in the helical domain codons E545K and E542K. Overall, 18% (11/62) of the *PIK3CA* mutations had occurred in the kinase domains and 82% in the helical domains. We did not detect the alteration E545A indicative for a polymorphism in the *PIK3CA* pseudogene of which the function is unknown [59]. In three primary tumors, two different *FGFR3* mutations were present (S249C together with A393E, G372C or R248C). One primary tumor contained two different *PIK3CA* mutations in the helical domains (E542K and E545K). There was no obvious co-occurrence or mutual exclusiveness between the different types of *RAS* and *PIK3CA* mutations.

**Table 1:** Frequencies of individual mutations in primary tumors of 257 patients.

*FGFR3* gene	N	*RAS* genes	N	*PIK3CA* gene	N
R248C	14	*HRAS* G12C	1	E542K	15
S249C	112	*HRAS* G12V	3	E545G	4
G372C	7	*HRAS* G13R	2	E545K	31
Y375C	27	*HRAS* Q61L	3	E545Q	1
A393E	1	*HRAS* Q61R	5	H1047L	4
K652M	2	*KRAS* G12C	3	H1047R	7
		*KRAS* G12D	7		
		*KRAS* G12V	3		
		*NRAS* Q61L	1		

The primary tumors were subsequently stratified into three subgroups based on stage and grade; low-grade NMI-BC tumors (pTaG1-2 and pT1G2), high-grade NMI-BC (pTaG3 and pT1G3), and muscle-invasive tumors (≥pT2). In Figure 4B–D, the distributions of *FGFR3*, *PIK3CA* and *RAS* mutations in these subgroups are illustrated. In the pTa-T1G1-2 group 88% of the primary tumors harbor a mutation in at least one of the five investigated oncogenes. Screening for *PIK3CA* and the three *RAS* genes increased the percentage mutant tumors with 10% when compared with *FGFR3* alone. In the grade 3 and muscle-invasive tumor groups, the total percentage of mutations in the oncogenes is much lower with 33% and 36%, respectively. In grade 3 tumors, the proportion of *RAS* mutations is relatively large, whereas *PIK3CA* mutations are more prominent in the muscle-invasive tumors. The addition of *PIK3CA* and *RAS* assays results in the detection of 13% additional mutant primary tumors in the grade 3 group and 15% in the muscle-invasive group.

### Co-occurrence of mutations

**Figure 5:**
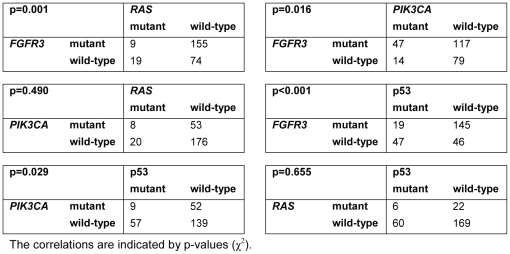
Relations between mutations in primary bladder tumors of 257 patients.

Of the 257 primary tumors, 26% had overexpression of p53, which is indicative of missense mutations. When we combine the oncogene mutations with those in the *TP53* tumor suppressor gene (Table 2), it appears that only 27 tumors (11%) were wild-type for all examined genes. There were 9 primary tumors with a co-occurrence of 3 alterations. There was a positive association of mutant *FGFR3* with *PIK3CA* mutations (p* = *0.016), with 77% of the *PIK3CA* mutations co-occurring with *FGFR3* (Figure 5). *FGFR3* mutations were strongly mutually exclusive with *RAS* mutations (p* = *0.001). Only 3.5% of the primary tumors contained a mutation in both genes. Interestingly, the mutual exclusiveness of *FGFR3* and *RAS* mutations remained significant in the subgroup of pTa/T1 G1/2 primary tumors, whereas *PIK3CA* and *FGFR3* mutations are significantly co-occurrent in grade 3 tumors. Both *FGFR3* and *PIK3CA* mutations were mutually exclusive with p53 overexpression (p*<*0.001 and p* = *0.029, respectively). *RAS* mutations were not mutually exclusive with *PIK3CA* and p53 mutations in the total cohort, nor in different tumor stage and grade subgroups.

**Table 2:** Combinations of mutations in primary bladder tumors of 257 patients.

Altered genes	Number	Percentage
*FGFR3*	97	37.7
*FGFR3* + p53	14	5.4
*FGFR3* + *PIK3CA*	39	15.2
*FGFR3* + *RAS*	6	2.3
*FGFR3* + p53 + *PIK3CA*	5	1.9
*FGFR3* + *RAS* + *PIK3CA*	3	1.2
*PIK3CA*	6	3.1
*RAS*	9	3.5
*RAS* + *PIK3CA*	4	1.6
p53 + *PIK3CA*	3	1.2
p53 + *RAS*	5	1.9
p53 + *RAS + PIK3CA*	1	0.4
p53	38	14.8
wild-type	27	10.5
**Total**	**257**	**100**

### Correlations of mutations with stage, grade

**Figure 6:**
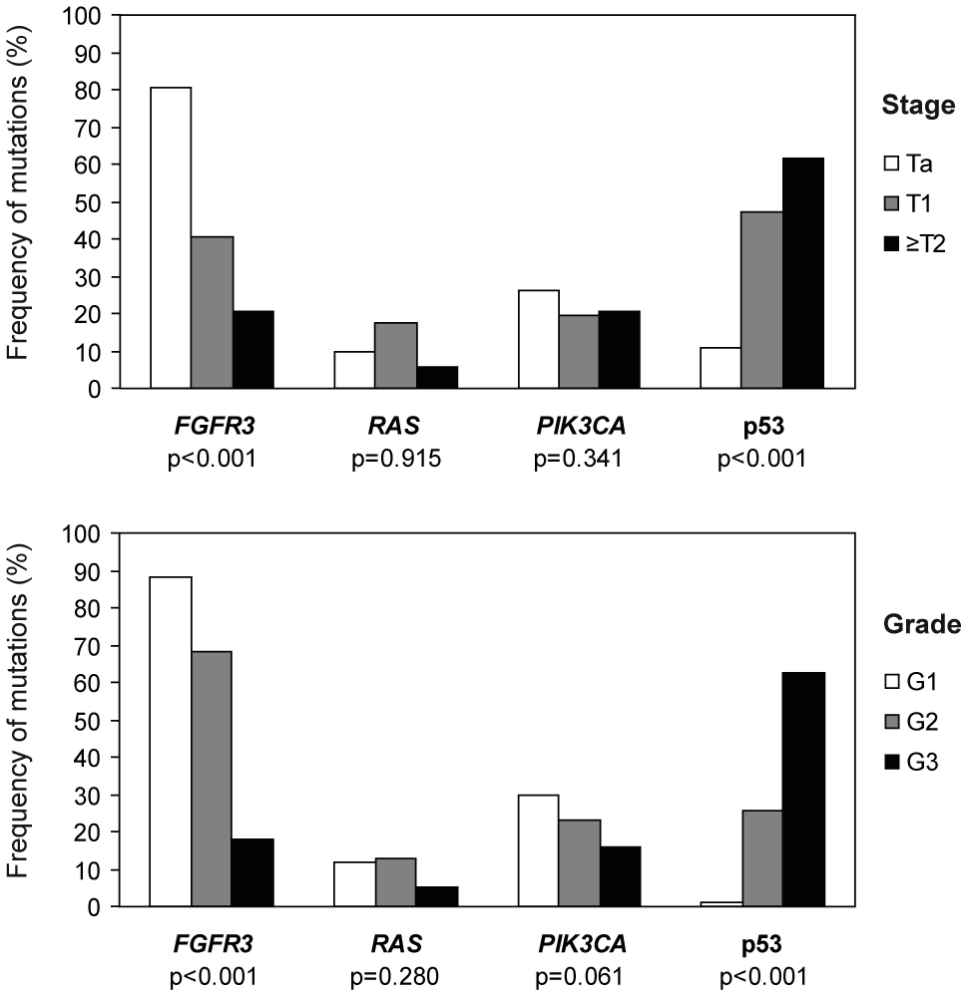
Frequencies of *FGFR3*, *RAS*, *PIK3CA* mutations and p53 overexpression according to stage and grade. The correlation of these alterations in primary bladder tumors of 257 patients with stage (A) or grade (B) is indicated by p-values (χ^2^).

We subsequently investigated the relation between stage and grade and the different mutations (Figure 6). In primary tumors there was a significant correlation of *FGFR3* with low stage and grade and a correlation of p53 overexpression with high stage and grade, as shown previously [39]. However, no significant association was observed between *RAS* mutation status and stage or grade. The distribution according to stage was 10% pTa (16 of 166), 18% pT1 (10 of 57), and 6% muscle-invasive tumors (2 of 34). Regarding *PIK3CA*, the prevalence of mutations was higher in low-grade tumors: 30% grade 1 (25 of 84), 23% grade 2 (27 of 117), and 16% grade 3 (9 of 56), however this association was not statistically significant (p = 0.061). No correlation with stage was observed.

### Prognostic value

Fifty-nine percent (154/257) of the patients in our study developed one or more recurrences, 10% had progression in stage and/or to grade 3, 19% died of disease. None of the investigated alterations in *FGFR3*, *RAS*, *PIK3CA* and p53 in the primary tumor was a predictor for development of a recurrence (recurrence-free survival p>0.05). Mutation frequency of *PIK3CA* in patients with recurrences was similar compared to patients without recurrences 24% (37/154) versus 23% (24/103). For *RAS* mutations, these frequencies were 12% and 10%. There was also no relation between the mutation status of *RAS* and *PIK3CA* and recurrence rate. As we showed previously, patients with an *FGFR3* mutant primary tumor have a lower risk of progression and a better disease-specific survival, whereas patients with p53 overexpression have high risk of progression and low disease-specific survival [32], [39]. However, *PIK3CA* or *RAS* mutations were not significantly associated with progression (p* = *0.129, p* = *0.694) or disease-specific survival (p* = *0.205, p* = *0.447) in the entire cohort, nor in different tumor stage and grade subgroups. Combining *RAS* and *PIK3CA* mutation status provided similar results. Furthermore, adding *RAS* or *PIK3CA* mutation status to *FGFR3* or p53 did not result in a better prediction of recurrence-free, progression-free or disease-specific survival compared to *FGFR3* or p53 alone. There were also no significant correlation of individual *RAS* isoforms and *PIK3CA* mutations in helical or kinase domains with stage, grade or recurrence-, progression-, and disease-specific survival. Furthermore, no significant correlation was found between *RAS* or *PIK3CA* mutations and altered Ki-67 (p = 0.413, p = 0.227) or p27^Kip1^ (p = 0.126 and p = 0.580) expression, markers indicative for a worse prognosis in bladder cancer [60], [61].

### Frequencies of mutations in recurrences

**Figure 7:**
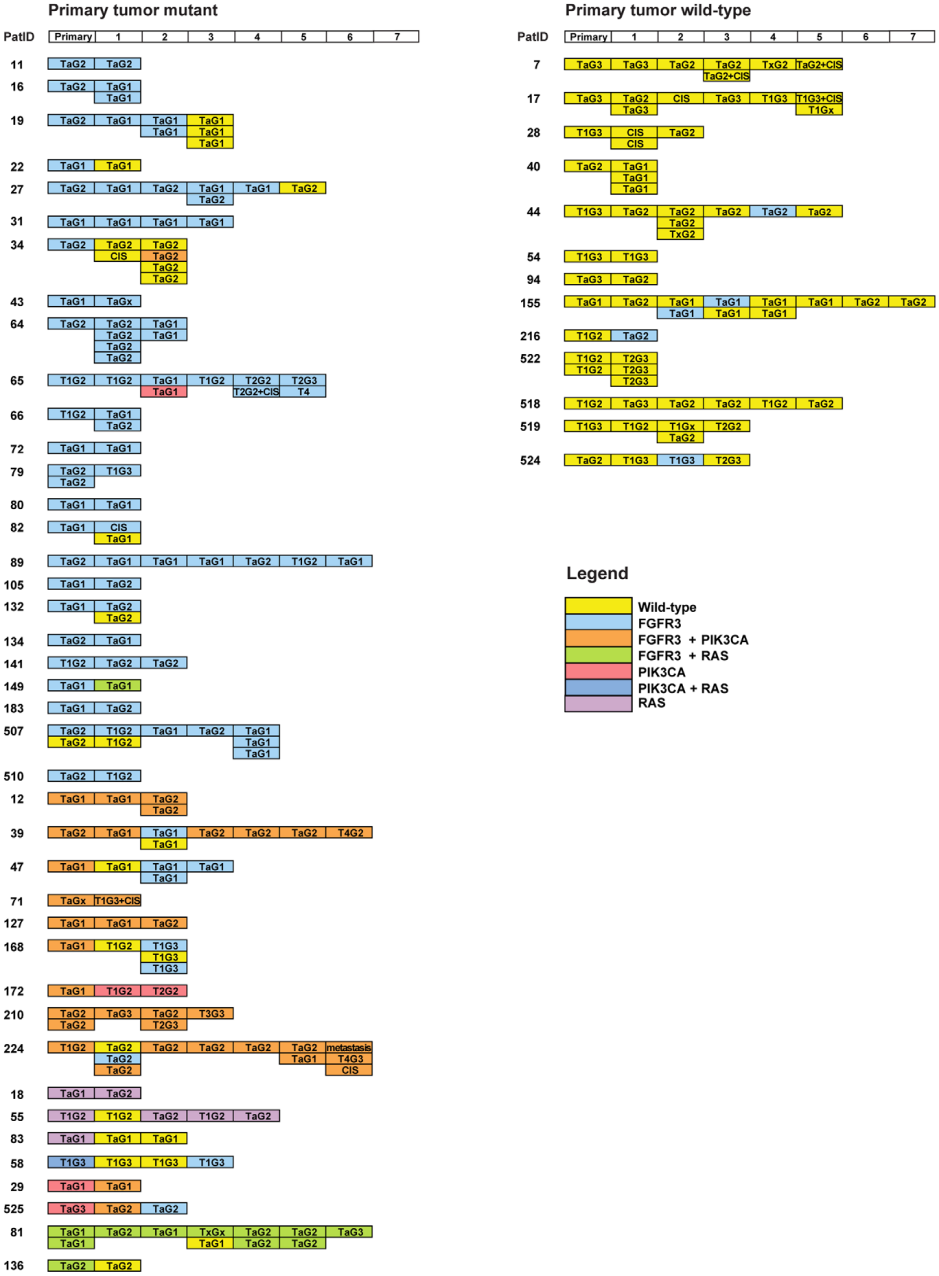
Detailed overview of the mutation status of 54 primary and 184 recurrent tumors. A: mutant primary tumors and their recurrences; B: wild-type primary tumors and their recurrences. The first column indicates the primary tumor. The successive boxes indicate temporally sequential recurrences removed in different transurethral resections (indicated by a sequence number on top). Multifocal tumors removed at the same transurethral resection are positioned underneath each other. Stage and grade of the tumors, mutation status (indicated by a color) and patient ID of the 54 patients is indicated.

From 54 patients that were treated at Erasmus MC and had developed one or more recurrences, tissue was available of 184 recurrences (including multifocal recurrences). Here, we wanted to investigate if mutation status persists in multiple recurrences of the same patients with the purpose to examine if it is valuable to start a future longitudinal study on surveillance with the mutation assays by analyzing urine samples. We only examined mutation status of the genes for which we have developed the SNaPshot based mutation assay (i.e. *FGFR3*, *PIK3CA* and *RAS*). P53 overexpression was not determined in recurrences. The frequency of p53 overexpression was also low (6/54) in the primary tumors of this group of patients consisting mainly of NMI-BC tumors. A detailed overview of stage, grade and mutation status of these tumors is presented in Figure 7. In patients with a wild-type primary tumor, recurrences were mostly wild-type (49/54 recurrences), while 5 harbored an *FGFR3* mutation. One recurrent tumor contained two different *PIK3CA* mutations (E542K and E545K). Interestingly, in recurrences *PIK3CA* mutations in addition to an *FGFR3* mutation was associated with higher grade compared to recurrences harboring an *FGFR3* mutation alone (p = 0.012). If we stratify for patients with a mutant primary tumor, 81% of the recurrences were also mutant and the individual frequencies were 75% (98/130) for *FGFR3*, 23% (30/130) for *PIK3CA*, and 10% (13/130) for *RAS*. Interestingly, there was a 100% consistency in the type of mutation for *RAS* and *PIK3CA* among different tumors of the same patient. We earlier observed that some recurrences were wild-type when the primary tumor was mutant for *FGFR3* [17]. In the present study, there were 20 of 130 recurrences (15%) in the patient subgroup with a mutant primary tumor that had progressed to grade 3, CIS or muscle-invasive bladder cancer (Figure 7). Of these, 90% (18/20) were mutant and therefore could be detected with the mutation assay. The wild-type recurrences in this patient group do not progress more often than the mutant recurrences; 8% (2/25) of the wild-type recurrences had progressed to CIS and to grade 3 (Figure 7), compared to 17% mutant recurrences. One of these wild-type recurrences co-occurred together with two mutant tumors. We further determined the time point at which the wild-type recurrences occurred during follow-up. Most of the wild-type recurrences (18 of 25) co-occurred together with a mutant recurrence or were later followed by a mutant recurrence, whereas 7 occurred as wild-type alone at the end of the follow-up period when no further data was available.

**Figure 8:**
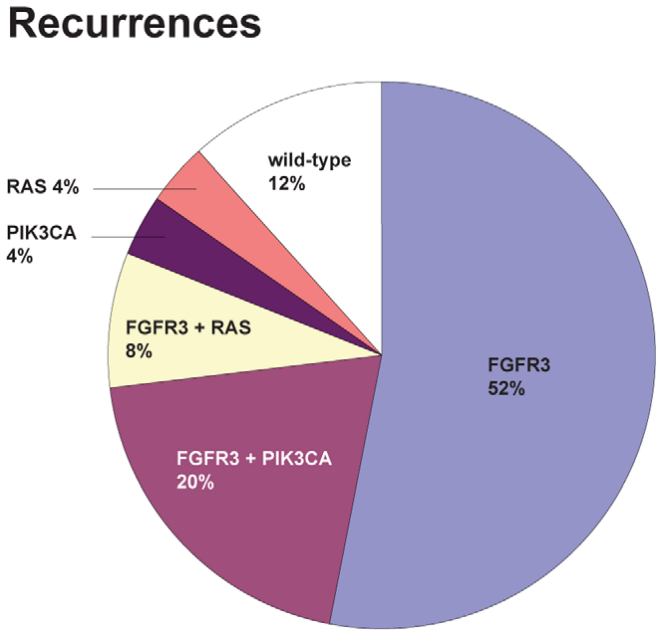
Frequency of mutations in recurrence events of patients with a mutant pTa/T1G1/2 primary bladder tumor. Frequency of *FGFR3*, *RAS*, and *PIK3CA* mutations is indicated.

One of the purposes of this study was to investigate if the mutation assays are a potential tool for the detection of recurrences in order to reduce the number of cystoscopical examinations and whether it is useful to initiate a large longitudinal study with these mutation assays for detection of recurrent bladder tumors using DNA extracted from urinary cells. Patients that are eligible for such a follow-up are those that present with a mutant pTaG1-2 or pT1G2 primary tumor. For this subgroup of patients the frequency of mutations in the *FGFR3*, *PIK3CA* and *RAS* genes when counted per recurrence event (i.e. in case of multiple tumors removed at transurethral resection, mutation data were combined) are illustrated in Figure 8. The figure shows that in this group of patients a mutation is present in 88% of the recurrence events. This is an increase of 8% when compared to *FGFR3* alone.

## Discussion

Activating point mutations in oncogenes present excellent biomarkers for diagnostic assays and targets for therapy. In urothelial tumors somatic mutations in the *FGFR3*, *HRAS*, *NRAS*, *KRAS* and *PIK3CA* genes may be of use for early detection of primary and recurrent tumors in urine-based assays, for prognosis prediction, and as a companion diagnostic for targeted therapies. In order to facilitate the detection of RAS mutations, we first designed an assay that simultaneously investigates 19 possible mutations in 10 codons of the 3 *RAS* genes. We used this bladder cancer specific RAS-BC assay together with similar assays that we developed previously for *FGFR3* and *PIK3CA* [53], [55], to investigate the frequency of these mutations in an unselected series of primary tumors of 257 patients representing all stages and grades and 184 successive recurrent bladder tumors of 54 patients. The frequency of *RAS* mutations in our study is similar to that reported by others with different techniques [41], [42]. *KRAS* and *HRAS* mutations occurred with equal frequency. *NRAS* mutations were not frequent in bladder cancer.

One of the main problems to address in bladder cancer is the high recurrence rate and the need for efficient markers to detect recurrences in a non-invasive way. Screening for the presence of recurrences using urine-based assays can potentially improve quality-of-life and reduce costs [19], [20], [21]. The SNaPshot based mutation assays that we developed might be useful particularly for urine analysis where only small amounts of DNA can be isolated and the percentage of non-tumor cells may vary [53]. The assays are also easy to perform, 100% reproducible, and inexpensive (material costs amount under 10 dollar per analysis [56]). Furthermore, the assays produce a positive signal, are easy to interpret and interobserver agreement is very high. Therefore, they are a suitable candidate for clinical implementation. We have previously shown that *FGFR3* mutation analysis on urine samples from bladder cancer patients was able to detect recurrent tumors [32], [53], [54]. Here we first investigated the frequency of patients that could be eligible for follow-up based on mutation status of the primary tumor. Furthermore, we investigated whether mutation status is consistent in recurrent tumors of a patient with the purpose to examine if it is useful to start a study on surveillance with these mutation assays by analyzing urine samples in a large longitudinal study. If the frequency of these mutations in recurrences is low, it would not be useful to initiate such a study.

The addition of the *RAS* and *PIK3CA* assays increases the percentage of low-grade NMI-BC patients to 88% for whom a surveillance scheme that includes mutation analysis on urinary cells could be of benefit. To determine whether mutation status is consistent in recurrences, we further screened 184 successive recurrences of 54 patients. In 88% of the transurethral resections performed during follow-up, one or more recurrences were mutant. Interestingly, there was a 100% consistency in the type of mutation for *RAS* and *PIK3CA* among different tumors of the same patient, which is in agreement with that the majority of recurrences are considered to be clonally related [62], [63], [64]. This homogeneity might be helpful in surveillance and therapy. However, in 12% of the follow-up assays the recurrence could not be detected with these assays. Nevertheless, the wild-type tumors in a patient with a mutant primary tumor do not progress very often and most of these wild-type tumors are later followed by a mutant tumor. Hence, these wild-type recurrences could potentially be detected in a later follow-up moment. An advantage of the mutation assays is that with the assays it is possible to detect mutant recurrences in the ureter and renal pelvis that cannot be seen by cystoscopy as was shown for *FGFR3* [65]. Cystoscopies are often the standard to which the sensitivity of new urine based biomarkers are compared. However the sensitivity of standard white light cystoscopy is estimated to be 77–83% [66], [67]. Hence, for a future follow-up scheme a combination of frequent urine assays and a reduced number of cystoscopies should be investigated.

We further investigated the prognostic value in terms of recurrence-free, progression-free and disease-specific survival of the different mutations in primary tumors. In bladder cancer, *PIK3CA* mutations had previously been associated with low grade and stage tumors [40]. In our study *PIK3CA* mutations were equally frequent in pTa, pT1 and ≥pT2 tumors, however the correlation of *PIK3CA* mutations with low grade was close to significance (p* = *0.061). There was no correlation between *RAS* mutations and stage and grade of the tumor. Our results on a large unselected series of consecutive tumors largely corroborate the data obtained by others [40], [41], [42], although their tumor panels were different, consisting of a relatively larger proportion of pTa tumors [40] or grade 3 tumors [41]. In contrast to *FGFR3* and p53 alterations, mutations in the *RAS* and *PIK3CA* genes were not predictors for recurrence-free, progression-free and disease-specific survival. There was also no difference in disease-specific survival for *RAS* and *PIK3CA* mutations between invasive and non-invasive groups.

The RAS-MAPK pathway and PI3K-Akt pathway are the two most important molecular pathways involved in cell growth in urothelial tumorigenesis [68], [69]. Cross-talk between the two signaling pathways can occur at several points and downstream they may converge on mammalian target of rapamycin kinase [70], [71]. RAS proteins are able to activate Phosphatidylinositol 3 kinase (PI3K) through a direct interaction with p110α of *PIK3CA* [72], [73]. In activating p110α, HRAS has been shown to be the most effective RAS isoform [74], [75]. Oncogenic activation of *RAS* genes can activate both Mitogen-activated protein kinases (MAPK) and PI3K pathways [76]. In addition to RAS, upstream FGFR3 is also able to activate both pathways. *FGFR3* mutations were mutually exclusive with *RAS* mutations in accordance with their signaling through the same pathway in bladder cancer [37]. Interestingly, *PIK3CA* mutations generally co-occur with *FGFR3* mutations suggesting an additive oncogenic effect of *PIK3CA* to *FGFR3* mutations. In our study, primary tumors harboring a *PIK3CA* mutation in addition to an *FGFR3* mutation were not different in stage or grade compared to those containing an *FGFR3* mutation alone. However, recurrences carrying both mutations were significantly higher in grade.

There is accumulating evidence that the three different *RAS* isoforms and helical and kinase domains of *PIK3CA* comprise different functions [77], [78], [79], which also might explain the tissue specific frequency of mutations. Recent functional assays showed that, the helical domain mutant of *PIK3CA* can be activated by RAS while the kinase domain mutant is not dependent on RAS binding [77], [79]. In breast cancer, mutations in the kinase domain are of better prognostic value than mutations in the helical domain, which might be explained by this synergy of RAS with oncogenic helical domain of *PIK3CA*. We therefore compared specific mutations in *RAS* isoforms and *PIK3CA* domains in relation to prognostic factors. However, in our study mutations in *RAS* isoforms and *PIK3CA* helical or kinase domains were not significantly correlated with different stage and grade or recurrence-free, progression-free, and disease-specific survival. There was also no difference in frequency of mutations that co-occurred with *RAS* mutations between helical and kinase domains of *PIK3CA*.

FGFR3 targeted therapy is being considered for muscle-invasive bladder tumors and recently a Phase II study has initiated in patients with advanced urothelial cancer (NCT00790426). *FGFR3* mutations are present in 21% of the MI-BC, and it was reported that overexpression of the receptor occurs in almost 40% of MI-BC [80]. This suggest that FGFR3 targeted therapy could be useful for about half of the MI-BC patients. The assays presented in this work could serve as a companion diagnostic to select patients for such a therapy since mutations in the *RAS* and *PIK3CA* genes, together amounting to 27% in MI-BC, might prohibit the effect of FGFR3 inhibitors. For example in pre-clinical studies of multiple myeloma, tumor cells are resistant to inhibition of the Fibroblast Growth Factor Receptor 3 (FGFR3) in the presence of a RAS mutation [12], [13]. The assays may also be useful for future therapies targeting the epidermal growth factor receptor (EGFR) in bladder cancer, which are currently tested in clinical trials. For advanced colorectal and lung cancers, patients currently are screened for mutations in the *KRAS* gene as therapy targeting EGFR is not effective when these tumors harbor mutations in the pathway downstream of EGFR [10]. Because of the molecular heterogeneity of bladder cancers, optimal targeted therapy will require the combined use of inhibitors targeting multiple molecular pathways [81], [82]. With the current development of small molecule inhibitors targeting receptor tyrosine kinases in the MAPK and PI3K pathways, the detection of mutations will become increasingly important to stratify patients.

The data presented here suggest that surveillance by mutation analysis for *FGFR3*, *PIK3CA* and the *RAS* genes in combination with extension of the period between cystoscopies could be a useful follow-up strategy for those patients presenting with a mutant NMI-BC, grade 1–2 primary tumor. However, the true value of the mutation assays as biomarker for the detection of recurrent bladder cancer in voided urine samples needs to be established in a longitudinal study on patients under surveillance for recurrent disease. The mutation assays may further be useful as a companion diagnostic to define patients with MI-BC who may benefit from therapies targeting FGFR3 or other receptors and downstream targets.
